# Conservative Follow-up of Severely Displaced Distal Radial Metaphyseal Fractures in Children

**DOI:** 10.7759/cureus.3259

**Published:** 2018-09-05

**Authors:** Deniz Akar, Cenk Köroğlu, Serkan Erkus, Ali Turgut, Önder Kalenderer

**Affiliations:** 1 Department of Orthopaedics and Traumatology, Milas State Hospital, İzmir, TUR; 2 Department of Orthopaedics and Traumatology, Ardahan State Hospital, Ardahan, TUR; 3 Department of Orthopaedics and Traumatology, Tepecik Training and Research Hospital, Izmir, TUR; 4 Department of Orthopaedics and Traumatology, Tepecik Training and Research Hospital, Istanbul, TUR; 5 Department of Orthopaedics and Traumatology, Tepecik Training and Research Hospital, İzmir, TUR

**Keywords:** distal radial fractures, remodeling, conservative treatment, children

## Abstract

Introduction

Distal radius fractures are the most frequent fractures seen in pediatric population and usually treated with closed reduction and casting. However, there is a risk of reduction loss and/or angulations in distal radial metaphyseal fractures. The purpose of this study is to evaluate the radiological and functional results of pediatric patients with distal radius metaphyseal fractures in which excessive displacement and/or angulations were accepted and to question upper acceptable limits in light of current literature.

Methods

Patients between five and 15 years of age with displaced distal radius fractures who were treated conservatively with significant angulation or translation were included in this study. Patients’ demographic data were gathered from hospital’s digital database. Clinical and radiological evaluations of all patients were done prospectively based on the last outpatient clinic control. Range of motion of wrist and elbow joint was measured with a goniometry, neurovascular status was documented, muscle strength was assessed and finally existing deformity measurements were performed clinically. Radiological evaluation was performed on pre-reduction, post-reduction, cast removal, 6th and 12th months and final examination radiographs. All measured values were compared with uninjured side. Radiologically, the percentage of translation, the amount of angulations, the distance from the fracture to the epiphyseal line, and the radius lengths were measured. Radial inclination and palmar tilt angles as well as ulnar variance and residual angulation were measured in both antero-posterior (AP) and lateral forearm radiographs. The Mann–Whitney U test was used to compare the variables in SPSS version 21. p < 0.05 was considered statistically significant.

Results

Twenty-nine patients with a mean age of 8.8 ± 3.1 years were included in this study. The mean follow-up duration was 17.4 ± 6.7 months. Compared to the uninjured side, in 24 (83%) patients, there were no limitations on wrist movements except five patients in forearm pronation clinically. In patients with re-displacement, the mean displacement occurrence time was 13.3 ± 4.9 (7–21) days. The translational and/or angulations in AP and lateral radiographs fully remodeled at the end of 6th month.

Conclusion

This study demonstrates that radial and dorsal angular deformities up to 39° and 22° volar angulation and complete displacement correct fully in children up to 10 years old. In children between 10 and 15 years, the dorsal angulation up to 38°, radial angulation up to 23°, and volar angulation up to 16° are acceptable for remodeling capacity of the child.

## Introduction

Distal radius fractures are the most frequent fractures seen in pediatric population (20.2%) and constitute approximately 1/6 of the fractures treated in emergency department. The distal radial physis and distal ulnar physis are responsible for 75 to 80% and 20% of the longitudinal growth, respectively [1,2]. The rapid growth feature increases fracture tendency at the lower end of the radius, because distal metaphysis is relatively weak due to continuous remodeling. Fractures are seen especially in pubertal growth ages (11–14 years in males, 8–11 years in females) and in spring and summer months when physical activity increases [3,4]. Displaced distal radius fractures are usually treated with closed reduction and casting [5]. Prevention of reduction loss is the main issue in conservative treatment [6].

The treatment of pediatric patients with an angulated distal radius fracture due to reasons such as late presentation or malpositioned healing fracture is unclear. Because they will result in possible functional impairment if not align, some authors suggest recurrent closed reduction maneuvers because of possible functional impairment [7,8]. Conversely, some authors report that accepting at the current position without additional maneuver is proper approach in order to avoid potential physeal injury. They suggest waiting until skeletal maturity and then performing corrective osteotomy if it is still necessary [1,2].

Orthopedic surgeons are prone to perform more aggressive treatment modalities in pediatric patients’ fractures nowadays. Acceptance limits for conservative treatments are decreasing and surgical indications are expanding. The purpose of this study is to evaluate the radiological and functional results of pediatric patients with distal radius metaphyseal fractures in which excessive displacement and/or angulation was accepted and to question upper acceptable limits in light of the current literature.

## Materials and methods

Pediatric patients with distal radius metaphyseal fractures treated in authors' institution between 2012 and 2014 were retrospectively reviewed. Institutional review board approved the study protocol and this study was carried out in accordance with the ethical standards laid down in the 1964 Declaration of Helsinki and its later amendments. Patients between 5 and 15 years of age with displaced distal radius fractures who were treated conservatively with significant angulation or translation were included in this study. The reasons for acceptance of displacement were: patients with systemic diseases such as upper respiratory tract infections which are contraindication for general anesthesia, occurrence of reduction loss with late presentation and finally patients with neglected injuries who were referred from other hospitals. Patients who were followed less than one year and patients who rejected to participate in the study were excluded.

Patients’ demographic data were gathered from hospital’s digital database. Radiological analysis was performed by using Picture Archiving and Communication System (PACS). Clinical and radiological evaluations of all patients were done prospectively based on the last outpatient clinic control. All cases were treated with long arm plaster after reduction. In all cases, long arm casts were changed to short arm form after three to four weeks according to radiological fracture healing findings (callus formation occurrence in at least two cortices). Once the cast was removed, wrist and elbow movements started. It was suggested to avoid sports activities for three months.

**Figure 1:**
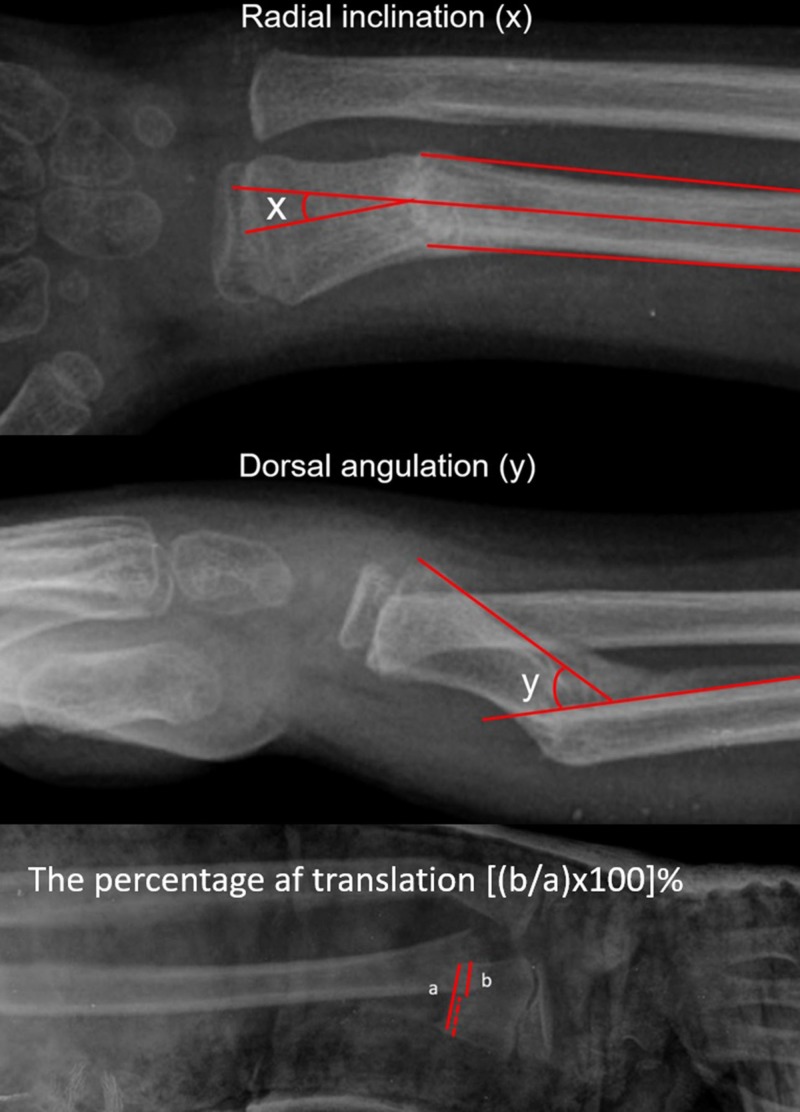
Figures demonstrating the method of measurements of angulations and translation. (a) Measurement of radial inclination, (b) dorsal angulation and (c) the percentage of translation.

As a standard protocol, the cases were invited to the outpatient clinic for the radiological control in every week during the first month, at the time of cast removal and at six months intervals afterwards. After removal of the cast, range of motion of wrist and elbow joints was measured with a goniometry, neurovascular status was documented, muscle strength was assessed and finally existing deformity measurements were performed clinically. Radiological evaluation was performed on pre-reduction, post-reduction, cast removal, sixth and twelfth months and final examination radiographs. All measured values were compared with uninjured side. Radiologically, the percentage of translation, the amount of angulations, the distance from the fracture to the epiphyseal line, and the radius lengths were measured. Radial inclination and palmar tilt angles as well as ulnar variance and residual angulations were measured in both anterior-posterior (AP) and lateral forearm radiographs (Figure 1).

**Figure 2:**
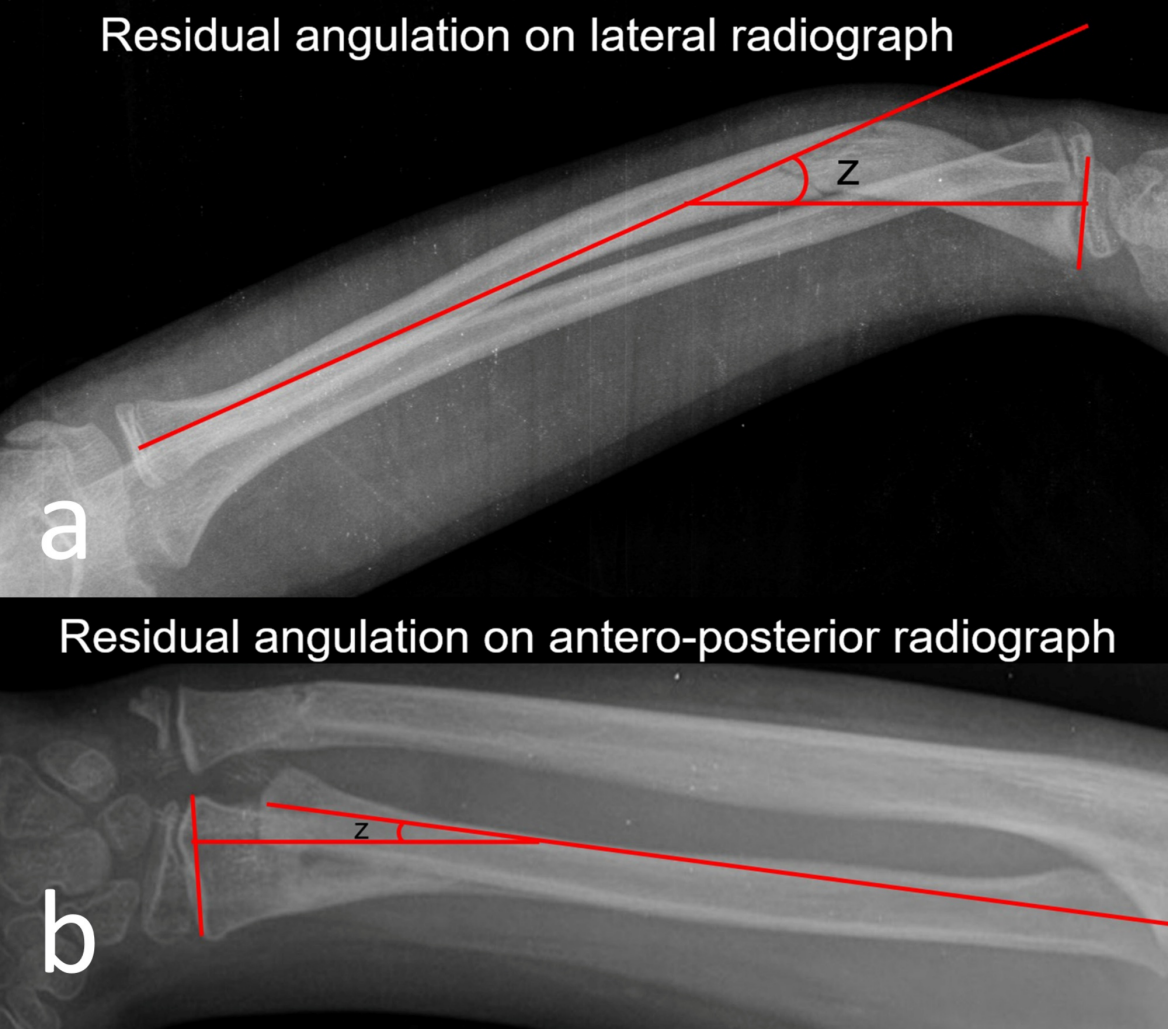
References about measurement of residual angulations. (a) Measurement of residual angulation on lateral radiograph, and (b) residual angulation on anteroposterior radiograph.

After removal of the cast, the residual angulations were measured in accordance with the distance of the long axis of the proximal fracture fragment perpendicular to the radial epiphyseal line (Figure 2). The amount of fracture angulations was measured by an independent trauma surgeon.

## Results

The mean age of the patients was 8.8 ± 3.1 (5–15) years, 21 (72%) of them were male and eight (28%) were female. The mean follow-up period was 17.4 ± 6.7 (12–30) months. The mean casting time was 6.5 ± 0.7 (5–8) weeks. Long arm cast was applied meanly 3.9 ± 0.4 (3–5) weeks, and short arm cast 2.6 ± 0.5 (2–4) weeks.

Eighteen patients had isolated distal radius fractures and 11 had (38%) associated distal ulna fracture. Thirteen fractures (45%) were on the dominant side and 16 fractures (55%) were on the non-dominant side. No complaints of pain were observed in any patient. All patients were able to perform daily and sportive activities without any problems. Parents were satisfied with the results. Compared to the healthy side, in 24 (83%) patients, there were no limitations on wrist movements. Nonetheless, in two cases (7%) and in three cases (10%), 5° and 10° of pronation limitation was detected, respectively. In patients with re-displacement, the mean displacement occurrence time was 13.3 ± 4.9 (7–21) days.

**Figure 3:**
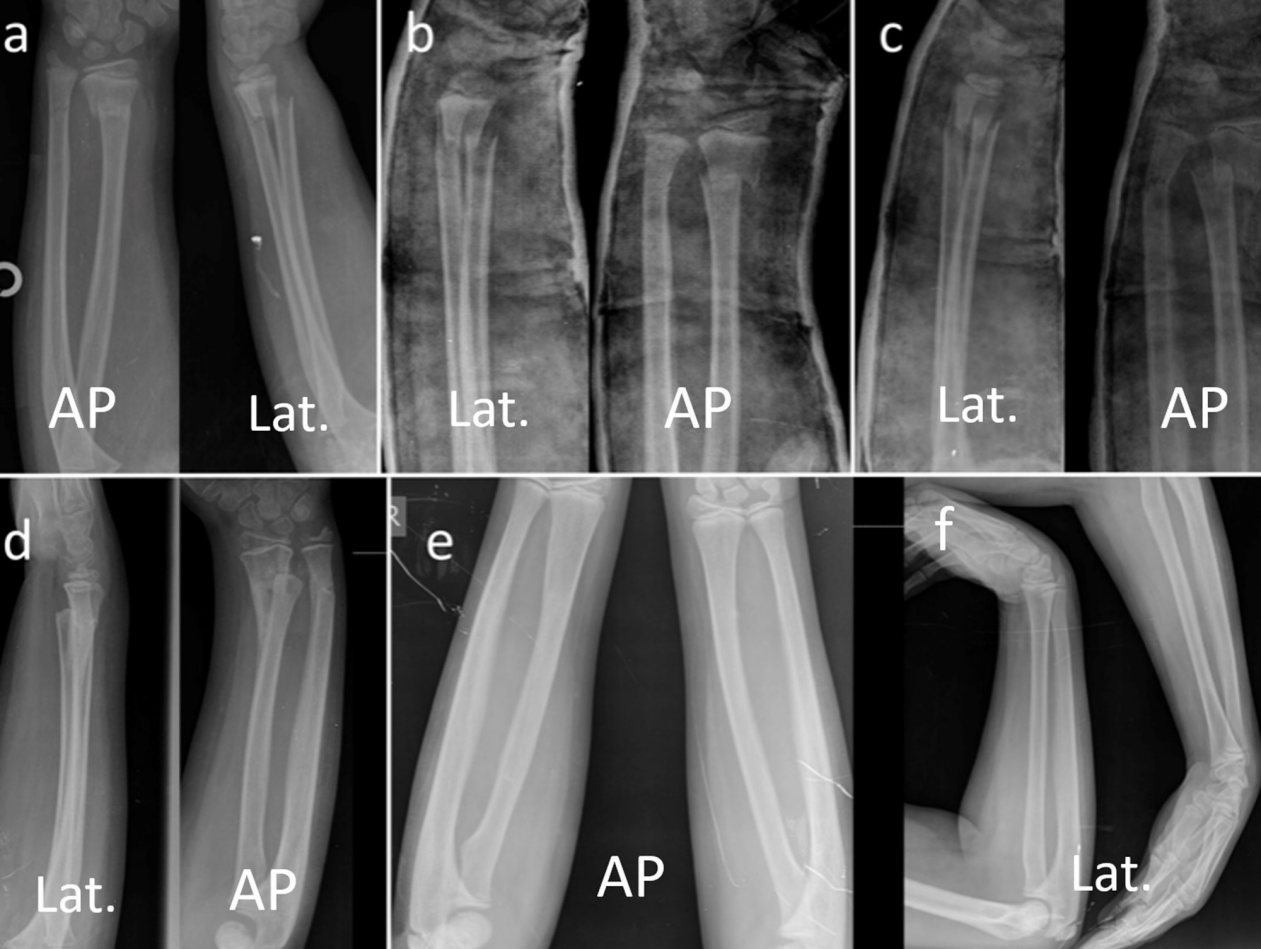
Case #1. Serial radiographic examination of seven-year-old girl with distal radial metaphyseal fracture. (a) Initial radiograph on admission. (b) Immediate closed reduction, the angulations were in acceptable range in both planes. (c) Re-displacement in cast. (d) Radiographs after the cast removal. (e,f) Final comparison radiographs with the un-injured side at 30th month showing full remodeling and normal alignment. AP: Antero-posterior; Lat.: Lateral.

**Figure 4:**
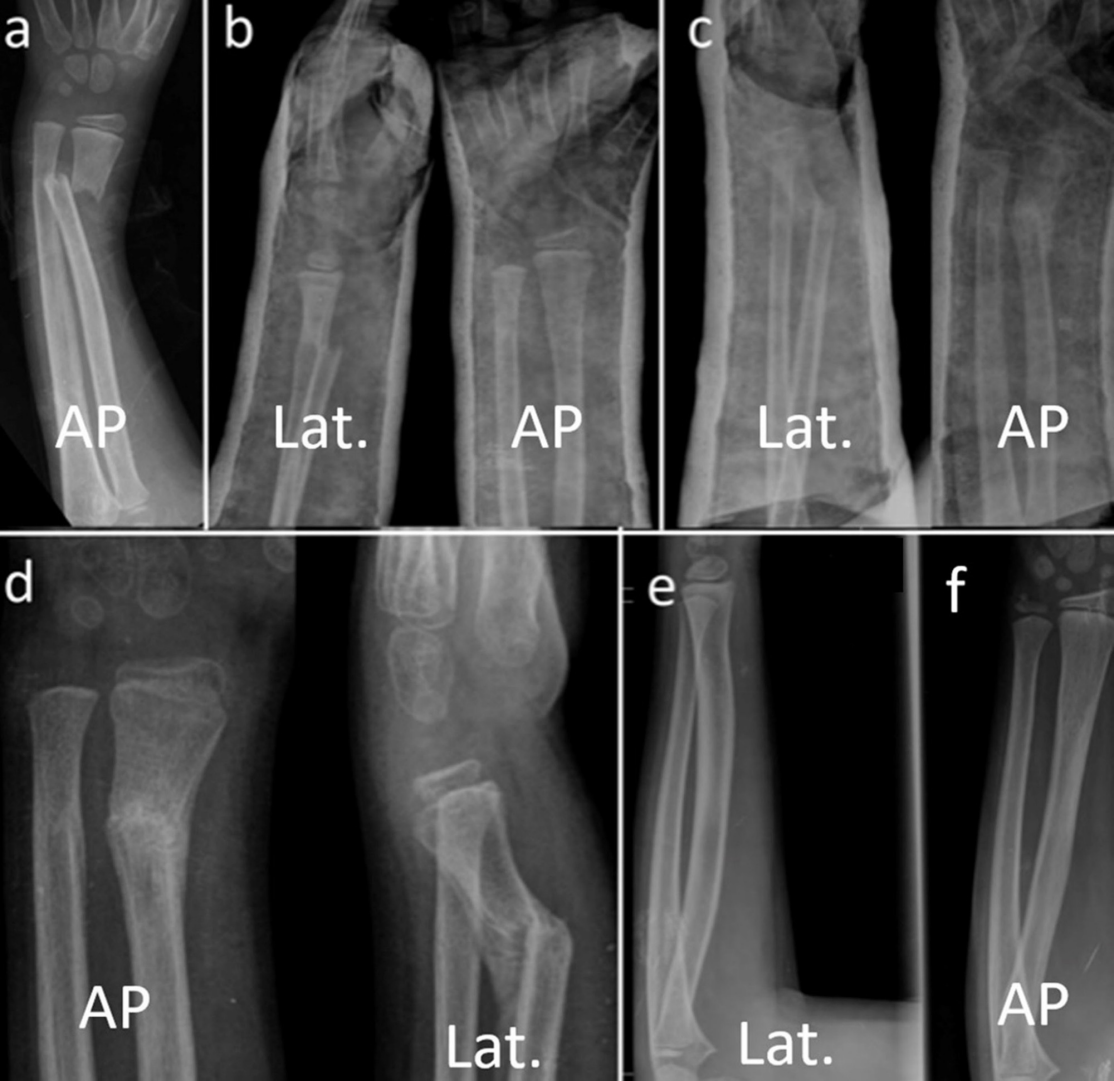
Case #2. Serial radiographic examination of eight-year-old girl with distal radial metaphyseal fracture. (a) Initial radiograph on admission. (b) Immediate closed reduction, the angulations were in acceptable range in both planes. (c) Re-displacement in cast. (d) Radiographs after the cast removal. (e,f) Final radiographs at 30th month showing full remodeling and normal alignment. AP: Anteroposterior; Lat.: Lateral.

The mean radial-ulnar angulation on the AP radiographs of 16 patients was 20.5 ± 8.5 (8°–39°) when the cast was removed, while the final angle decreased to 0.25 ± 1 (0°–4°) (p < 0.01). The mean amount of radial angulation in the lateral radiographs of 27 patients was 25 ± 8.9 (5°–45°) when cast was removed. At the last follow-up, this angulation was measured as 3.7 ± 4.2 (0°–12°) (p < 0.01). The translational amount in AP and lateral radiographs was 54% ± 31% (10–100%) at the time of cast removal, 26.3% ± 14.3% (10–60%) at three months and fully remodeled at the end of sixth month (p < 0.01). It was observed that the translations on AP and lateral radiographs of all patients were completely remodeled at the sixth month (p < 0.01) (Table 1) (Figures 3, 4).

**Table 1:** Remodeling of angular and translational deformities.

	At the time of cast removal	Difference between cast removal – 3rd month ° (p)	Difference between 3rd month – 6th month ° (p)	Difference between 6th month – final control ° (p)	Difference between cast removal – final control ° (p)
Coronal plane angulation (n:16)	20.5 ± 8.5 (8–39)	5.7° ± 3.39 (2–12) (p: 0.04)	5.9° ± 2.77 (2–12) (p < 0.01)	4.65° ± 3.34 (2–13) (p < 0.01)	16.25° ± 7.57 (8–33) (p < 0.01)
Sagittal plane angulation (n:27)	25 ± 8.9 (5–39)	7.5° ± 3.72 (2–15) (p < 0.01)	6.8° ± 3.26 (2–16) (p < 0.01)	4.8° ± 3.39 (1–13) (p < 0.01)	19.1° ± 8.62 (0–32) (p < 0.01)
Translation in any plane (n:20)	54% ± 31.01 (10–100) (p < 0.01)	26.3% ± 14.33 (10–​​​​​​​60) (p < 0.01)	0	0	0

## Discussion

Distal 1/3 fractures of the forearm constitute 20% of all pediatric fractures [1,2]. In children, closed reduction and casting are accepted as a fundamental approach in the treatment of distal radius fractures [1,2,6]. The distal physis of radius is responsible for the 75–80% of longitudinal growth which closes between the ages of 14 and 16. It has been stated that if the distal physis of radius is not injured, very prominent angles and displacements can be corrected [9]. As a result, the extent of acceptance of the deformities within the cast is also widened, especially since axial deformations have spontaneous recovery potential [6,10-12].

There are different opinions about the quality of reduction and how much angulation can be accepted in distal 1/3 radius fractures. Under the age of 10, it has been stated that 10° to 50° sagittal plane angulation and 10° to 40° coronal plane angulation can be accepted [1,2,6,7,9,13-17]. In distal radius fractures, the data of the studies as to the acceptable deformity limits are given in Table 2.

**Table 2:** Publications about acceptable angulation limits.

Distal one-third fractures	Acceptable angular deformities (years: y)	
Fuller and McCullough, 1979[16]		20° (<14 y)
Larsen et al., 1988[14]		28° angulation (≤11 y)
Roy, 1989[15]		16° radial deviation 20° dorsal angulation
Wilkins and O’Brien, 1996[1]		30°–35° (sagittal plane)
Zimmermann et al., 2004[13]		10–​​​​​​​15° dorsal/volar angulation (<9 y)
Roth et al., 2014[17]		30° (<9 y)/25° (9-<12 y)/20° ≥ 12 y

Larsen et al. reported that the results of dorsal angulation of up to 20° and radial angulation up to 15° in children under nine years were good. They also reported that remodeling in children over 11 years of age was achieved by changing the orientation of the epiphysis plaque [14]. Wilkins-O'Brien indicated that 30–35 degree sagittal plane angulations would improve in children with a growth potential of at least five years [1].

Re-displacement rates following closed reduction in distal radius fractures have been reported to be 7–25% [12,18,19]. In pediatric population, fractures of the distal metaphyseal radius rarely cause functional deficiency because of high remodeling capacity. Significant improvements can be expected even in deformities which are not in main movement axis of the wrist such as radial deviation. In some studies, it has been reported that there is limitation in forearm rotation [7,8,20]. Roberts demonstrated that radial deviation of the distal fragment caused loss of forearm rotation [7]. They argued that this may be due to the narrowing of the interosseous space at fracture site. Högström et al. noted that there is a strong correlation between residual angular deformities and loss of forearm rotation [8]. In contrast, Nilsson and Obrant reported that forearm rotation loss is due to the initial displacement of the fracture, even anatomic reduction had been achieved after closed reduction [20].

Roberts compared the forearm motion ranges of both forearms in 50 healthy children without fractures and showed that forearm rotational movements may differ by up to 15° even in healthy individuals [7]. For this reason, they accepted the values above 15° of rotational loss as limitation in their work. In our study, there were no limitations on the motion of the wrist, except for the pronation. Pronation was limited in five patients. Limitations were 5° and 10° in two and three patients, respectively. Four of the five patients with limited pronation had residual dorsal angulations between 5° and 10° at their last follow-up. There was no radial angulation in any of the patients.

In a retrospectively designed study of 105 children with distal radius fractures, Jordan and Westacott reported that re-displacement is possible if optimal reduction (less than 10% translation and less than 10° angulation) could not be performed. Especially in patients with more than 50% translations, it is suggested that the possibility of re-displacement is elevated and therefore it is necessary to fix the fracture by a Kirschner wire in these patients [21]. The results of our study do not support the findings of aforementioned study. Even in patients with more severe displacement, the radius is remodeling and does not constitute an altered function.

Zimmermann et al. compared the fractures healed with volar and dorsal angulations. Mean follow-up of time was 10 years, it was reported that there was no difference between the two groups in terms of radiological improvement in patients with an average of 15 degrees volar and dorsal angulation. There was a significant level of supination limitation in patients with volar angulation. This was also attributed to the distal segment in pronation in the volar displaced fractures [13].

Zimmerman et al., in their study of 10 years of 220 patients with distal radius fracture treated conservatively, reported that residual deformity under 10 years did not affect the long-term outcome, and angular deformity above 20° and over half of the bone diameter adversely affected the outcome over the age of 10 years [22]. Roy reported that complete remodeling was achieved at displacements of 16° on the AP plane and 20° on the lateral plane, and there was no necessity for re-reduction maneuver for these patients [15]. Similarly, Hove and Brudvik reported that the results were excellent in 88 patients healed with deformity and conservative treatment was the gold standard for distal radius fractures [6]. In this study, all translations below and above 10 years of age improved clinically and radiologically. Neither patient had functional limitation.

Do et al. stated that 15° of angulation in any plane and up to 1 cm shortening were completely remodeled in 7.5 months in 34 re-displaced and angulated fractures. Functional impairment was not reported in any patients [23]. Short or long arm cast applications have been discussed in these fractures, but no difference was found in complications between the applications [24]. Despite the fact that all our cases were treated with long arm cast, re-displacement has occurred. We believe that re-displacement is not associated with above or below elbow casting.

There are some limitations to our study, of which the retrospective study design is probably the most important, and patient  s’ number is low.

## Conclusions

In conclusion, the present study demonstrates that radial and dorsal angular deformities up to 39° and 22° volar angulations and complete displacement correct fully in children up to 10 years old. In children between 10 and 15 years, the dorsal angulations up to 38°, radial angulations up to 23°, and volar angulations up to 16° are acceptable for remodeling capacity of the child.
